# Female partner preferences enhance offspring ability to survive an infection

**DOI:** 10.1186/1471-2148-14-14

**Published:** 2014-01-23

**Authors:** Shirley Raveh, Sanja Sutalo, Kerstin E Thonhauser, Michaela Thoß, Attila Hettyey, Friederike Winkelser, Dustin J Penn

**Affiliations:** 1 Konrad Lorenz Institute of Ethology, Department of Integrative Biology and Evolution, University of Veterinary Medicine Vienna, Savoyenstr. 1a, 1160 Vienna, Austria; 2 Present address: Department of Environmental Sciences Zoology and Evolution, University of Basel, Vesalgasse 1, 4051 Basel, Switzerland; 3 Present address: “Lendület” Evolutionary Ecology Research Group, Plant Protection Institute, Centre for Agricultural Research, Hungarian Academy of Sciences, Herman Ottó út 15, 1022 Budapest, Hungary

**Keywords:** Partner preference, Sexual selection, *Mus musculus musculus*, *Salmonella*, Pathogen clearance, Disease resistance, Pathogen-mediated sexual selection, Tolerance

## Abstract

**Background:**

It is often suggested that mate choice enhances offspring immune resistance to infectious diseases. To test this hypothesis, we conducted a study with wild-derived house mice (*Mus musculus musculus*) in which females were experimentally mated either with their preferred or non-preferred male, and their offspring were infected with a mouse pathogen, *Salmonella enterica* (serovar Typhimurium).

**Results:**

We found that offspring sired by preferred males were significantly more likely to survive the experimental infection compared to those sired by non-preferred males. We found no significant differences in the pathogen clearance or infection dynamics between the infected mice, suggesting that offspring from preferred males were better able to cope with infection and had improved tolerance rather than immune resistance.

**Conclusion:**

Our results provide the first direct experimental evidence within a single study that partner preferences enhance offspring resistance to infectious diseases.

## Background

Mating preferences are widespread and can function to confer indirect, genetic benefits for offspring [1-3]. Direct evidence for genetic benefits from mate choice come from studies that compare the fitness of offspring from females (experimentally) mated with preferred (P) versus non-preferred (NP) males (*experimental sexual selection)*[4-6]. For example, it has been shown that peahens (*Pavo cristatus*) had increased reproductive success when mated with P males [7,8]. Similarly, in house mice *(Mus musculus domesticus)* offspring sired by females’ P males had higher survival compared to offspring sired by NP males [9]. However, it remains unclear how mate choice enhances offspring survival. Since there is much genetic variation influencing immune resistance to infection [10-12], it has been suggested that mate choice may provide genetic benefits that enhance offspring resistance against infectious diseases (Hamilton-Zuk hypothesis) [13-15].

Since Hamilton and Zuk’s [13] classic paper, numerous studies have found that males with more parasites or weak immune responses have reduced secondary sexual traits compared to other males [16,17], and that females show preference for healthy versus experimentally parasitized males. For example, in house mice, males produce scent marks, which are attractive to females, and females are able to recognize and show preferences for the scent of healthy, uninfected versus experimentally infected males [18-21]. Such preferences may provide direct benefits (lower risk of disease transmission or increased paternal care) or indirect, genetic benefits [22,23]. For example, a study with stickleback fish (*Gasterosteus aculeatus*) found that offspring sired by more brightly coloured males were less likely to become infected after an experimental exposure to cestode larvae compared to duller males (though offspring of brightly coloured males also grew more slowly, they did not mount higher immune responses and they did not have improved hatching or post-hatch survival compared to dull males) [24]. Yet, there have been no studies to our knowledge that have experimentally tested whether female choice enhances offspring resistance to infectious diseases within a single study.

We conducted a study with wild-derived house mice (*Mus musculus musculus*) to test whether female preferences enhance offspring’s resistance to an experimental challenge of *Salmonella enterica* (serovar Typhimurium). Several genes control resistance to *Salmonella* in mice, which include MHC (major histocompatibility complex) [25], SLC11A1 (Ity/Lsh/Bcg/N-ramp1), and toll-like receptor 4 (Tlr4) [26-28]. After testing females’ preferences for individual males in dyadic ‘partner preference’ experiments, we mated females to either their P or NP males. The offspring of these pairings were experimentally infected with a mouse pathogen (*Salmonella enterica)* to assess their ability to resolve infection over three weeks (pathogen clearance). We expected that offspring sired by P males would have lower pathogen burdens (or loads) and enhanced survival compared to those sired by NP males. In addition, females can potentially enhance offspring survival due to differential *maternal investment* into offspring sired by high quality males (differential allocation hypothesis) [29-31]. Alternatively, it has been suggested that females should invest more into offspring when mating with low-quality males and enhance offspring body mass and survival (reproductive compensation hypothesis) [30,32-34]. Therefore, we also tested whether offspring from P sires had greater body mass compared to those from NP males.

## Results

### Reproduction

After the experimental matings with P or NP males, most females (83%) produced litters and females mated with a P male were more likely to produce a litter (14 litters from 15 pairings or 93% success) compared to females mated with NP males (11 litters from 15 pairings or 73%), though this difference was not significant (χ^2^ = 2.08, df = 1, p = 0.165). Females mated with P males produced significantly larger litters (mean ± SE = 7.2 ± 0.5) than those mated with NP males (mean ± SE = 6.3 ± 0.8) (df = 1, F _1, 12_ = 5.54, p = 0.036). In addition, heavier females produced larger litters than lighter dams (df = 1, F_1, 12_ = 5.48, p = 0.037; range: 2 to 11 offspring per litter). The interaction between sire treatment (P or NP) and dam body mass was non-significant (df = 1, F_1, 11_ = 0.91, *P* =0.656). No evidence was found to suggest that mean offspring body mass was affected by pairing dams with P or NP males (F _1, 11_ = 0.30, p = 0.594) or female body mass (F _1, 11_ = 0.16, *P* = 0.696), and the two-way interaction between partner preference and female weight was non-significant (F _1, 11_ = 0.23, p = 0.641). Pairing females with P males resulted in more male offspring compared to NP pairings and heavier females produced more sons compared to lighter females (pairing with P or NP male: Wald χ^2^ = 4.89, df = 1, p =0.027; female weight: Wald χ^2^ = 10.96, df = 1, p =0.001; interaction: Wald χ^2^ = 1.54, df = 1, p = 0.214).

### Offspring survival

**Figure 1:**
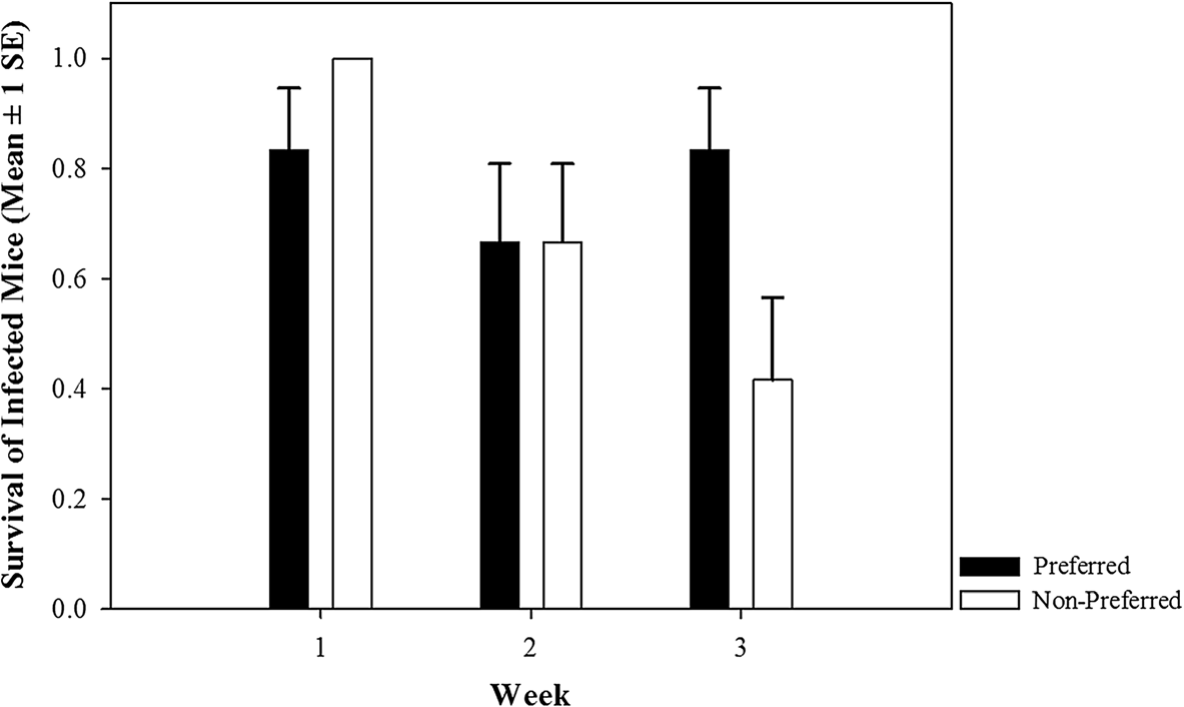
**Proportion of individuals that survived the experimental infection over the course of three weeks.** Black bars represent offspring sired by P and white bars sired by NP males. A significant interaction between partner preference treatment (P vs. NP) and week was found: survival between offspring from P and NP sires emerged three weeks after inoculation, with an enhanced survival of offspring sired by P males compared to young sired by NP males (see Table 1).

**Figure 2:**
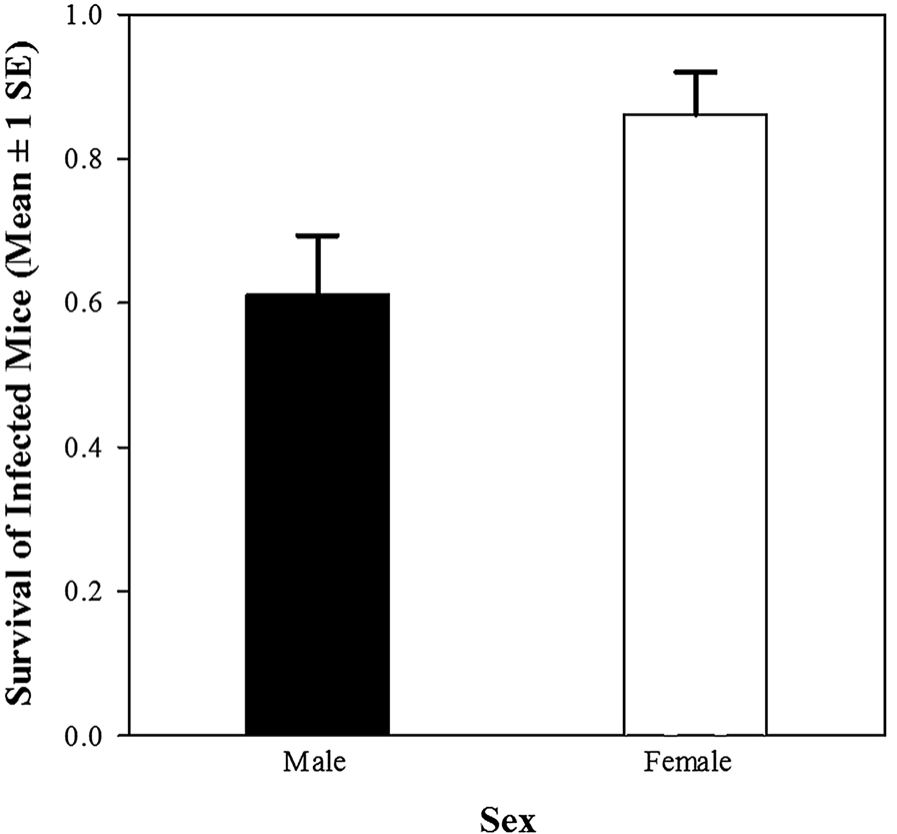
**Sex-dependent survival for females and males after the experimental infection.** Female offspring (n *=* 31, white bar) had an enhanced survival compared to male offspring (n *=* 22, black bar) regardless of the partner preference (see Table 1). Error bars represent standard error of the mean.

We challenged offspring with an experimental *Salmonella* infection and we found that 73% (53/72) of offspring survived the infection until euthanasia, and offspring sired by P males had higher survival than those of NP males (p *=* 0.0088, Table 1). Survival of offspring sired by NP, but not by P males decreased over time, as shown by the significant interaction between the partner preference treatment (P vs. NP) and week (p *=* 0.0016, Table 1; Figure 1). Female offspring (n *=* 31) had better survival than male offspring (n *=* 22) regardless of the partner preference (Table 1; Figure 2). The main effects of body mass, week, and all remaining two-way interactions did not affect survival (Table 1). Body mass of animals of P and NP sires used in the infection experiment did not differ significantly (*F*_1,68_ = 2.66; p = 0.11) or across weeks (*F*_1,68_ = 0.22; p = 0.64), yet males were heavier than females (males: 21.29 ± 0.54 g (mean ± SE); females: 18.95 ± 0.34 g; *F*_1,69_ = 15.36; p *<* 0.001). All two-way interactions were non-significant (all p > 0.15).

**Table 1:** Table 1 Effects of partner preference (P/NP), sex, week (1-3), and body mass on survival as calculated with starting date of the experiments and family ID as random effects using generalized linear mixed-effects modelling procedures

	**df**	**F**	***P***
Partner preference	40	7.595	0.0088**
Sex	26	12.569	0.0015**
Week	26	0.202	0.6565
Lgbodymass	25	0.101	0.7537
Partner preference ^x^ week	26	10.801	0.0029**
Sex ^x^ partner preference	24	2.510	0.1262
Sex ^x^ week	20	0.003	0.9545
Sex ^x^ lgbodymass	21	0.008	0.9298
Partner preference ^x^ lgbodymass	23	0.444	0.5118
Week ^x^ lgbodymass	21	0.062	0.8060

**: *P* < 0.01.

x: interaction between two variables.

### Offspring pathogen clearance

The overall pathogen load was unrelated to partner preference (P/NP), duration of infection (week), or sex (LMM, partner preference: *F*_1,35_ = 0.77, p *=* 0.38; sex: *F*_1,10_ = 0.05, p *=* 0.81; week: *F*_1,10_ = 1.27; p *=* 0.28). All two-way interactions remained non-significant (all p *>* 0.1).

## Discussion

We found that females experimentally mated to P males produced significantly more offspring (larger litters) than those mated to NP males, which confirms that non-random partner preferences confer fitness benefits [9]. Furthermore, we found that offspring sired by P males were significantly more likely to survive the experimental *Salmonella* infection than those sired by NP males. The difference in survival between offspring from P and NP sires emerged three weeks after inoculation, which corresponds precisely to the peak of *Salmonella* growth in the spleen of house mice [35]. We are confident that the differences in survival was due to the experimental infection because sham-infected mice never die in our experience [21,25,36], and the other offspring from our experimental matings, which were not experimentally infected (*N =* 108), all survived the three-week experiment. Our findings are consistent with a previous study that found outbred house mice are more likely to survive *Salmonella* infection than inbred mice [37]. Thus, our results support the hypothesis that female preferences can enhance offspring ability to survive infection.

The enhanced survival of offspring from P versus NP sires could have been due to differences in their ability to control pathogen burden (‘pathogen clearance’ or ‘immune resistance’) or their capacity to cope with or limit the damage caused by a given pathogen burden (‘disease resistance’ or ‘tolerance’) [38-40], or both. We found that pathogen loads did not differ between treatments, which suggest that female preferences enhanced tolerance to infection rather than immune resistance *per se*. However, as we were unable to assess the pathogen loads of the mice that died during the experiment, we cannot rule out possible benefits from controlling pathogen growth. Previous sexual selection (and ecoimmunology) studies have mainly measured immune responses to novel antigens (immunocompetence) or observed parasite burdens, but our findings emphasize the importance of using functional challenges with real pathogens and measuring hosts’ ability to cope with infection (health and survival) [20,38,41]. Otherwise, measuring only resistance to infection will fail to detect many important aspects of host defences, such as immunoregulation, which functions to minimize damage from immunopathology (‘optimal immunocompetence’ [20] or ‘optimal immunity’) [42-44]. Studies are needed to determine whether tolerance – like resistance to infection – is heritable [45,46], condition-dependent [47-50] and influences the expression of secondary sexual traits (e.g., scent or ultrasonic vocalizations in house mice). It is likely that females are able to obtain tolerant and disease resistant offspring by preferring males in overall good condition (rather than recognizing males with strong immune resistance per se) [44,51], as expected by the *genic capture hypothesis*[52,53].

The enhanced survival of offspring sired by P males may have been due to genetic effects, maternal allocation, or both. This benefit was potentially genetic, as it has been found that outbred mice have better survival following *Salmonella* infection compared to inbred (from sib-sib matings) mice [37]. Since neither litter size nor the offspring weight differed between P versus NP sires, we found no evidence for differential maternal allocation, though we cannot rule out this potential mechanism. We also found that females mated to P males produced more sons than those mated to NP males, though this bias in offspring sex-ratio could have been due to maternal or paternal effects. Heavier females produced significantly more sons than lighter females, suggesting offspring sex ratio depends on females’ condition, as predicted by the Trivers and Willard hypothesis [54]. Yet, differences in litter size and offspring sex ratio do not explain why P offspring had better survival compared to NP offspring, and therefore, it is more likely that this difference we found was due to genetic influences to disease resistance [28]. Further studies are needed to test whether genetic effects or differential maternal allocation can explain the variation in offspring survival after an infection.

Finally, female offspring had better survival following *Salmonella* infection compared to males, and as we found no sex differences in pathogen clearance, this result also appears to have been due to sex differences in tolerance to infection (this finding was not influenced by higher production of daughters by females mated with NP males, as animals were evenly allocated for partner preference and sex). Females generally have increased resistance to pathogens and parasites compared to males [55-57], and another study found that female mice showed lower *Salmonella* prevalence compared to males following experimental infection [37]. Such sex differences may be due to testosterone or other steroid hormones [58-60]. For example, it has been reported that estrogen administration in females resulted in a higher susceptibility to *Salmonella typhimurium*, whereas progesterone enhanced resistance to infection [61], though these findings are controversial [62]. Future studies are needed to compare sex differences in tolerance to *Salmonella* infection, and the underlying mechanisms, especially in wild or outbred house mice.

## Conclusions

Our findings provide the first direct experimental evidence to our knowledge that partner preferences enhance offspring survival following infection. We found no evidence that the offspring from preferred sires had more effective pathogen clearance than offspring from non-preferred sires, suggesting mate choice may enhance the ability to cope with infection (tolerance rather than pathogen resistance *per se*). Future studies are needed to investigate this hypothesis, and especially the underlying developmental mechanisms controlling tolerance to infection, both genetic and environmental maternal effects. Our results and the rapidly growing research on measuring tolerance to infection should inspire renewed interest on pathogen-mediated and sexual selection.

## Methods

### Study system

The mice used in this experiment were F2 descendants of wild-trapped house mice (*Mus musculus musculus*) from Vienna (48° 12’ 38” N; 16°16’54” E). The parental generation were trapped at 14 different locations within a 500 m radius and then bred between locations. F1 mice were tested in a social partner preference and either a female’s preferred (P) or the non-preferred (NP) male was assessed and paired accordingly (see below) [following 9]. The resulting offspring (F2 generation) were used for experimental infection (see below). All F2 mice were weaned at the age of 21 ± 1 days and were kept individually in standard mouse cages (26.5 × 20.7 × 14 cm) with wooden bedding (Abedd: aspen wood chips), enrichment material consisting of nesting material (Abedd: aspen wood shavings), and nest boxes. Food (Altromin rodent diet 1324) and water were provided *ad libitum*. A standard 12:12 h light cycle was maintained and temperature ranged from 22 to 25°C. After experimental infection animals were housed individually in ventilated cages (36.5 × 20.7 × 14 cm, IVC). All other housing conditions remained identical to conditions prior to infection.

### Social preference test

To test partner preference in wild-derived house mice, F1 mice were assigned to 30 triplets each containing one female (N = 30) and two unrelated males (N = 60). Prior to the social preference test, all females successfully mated and raised one litter to avoid any confounds from females’ sexual experience. Males were sexually mature (16.52 ± 0.35, mean ± SE), however they had no mating experience. On the day of the experiment, all mice were weighed. Mice were brought to the testing room at 12:00 to allow the animals to acclimate for six hours (five triplets were tested per day). During acclimation, male mice were stimulated with 5 μl of pooled female’s urine on filter paper (collected and mixed from seven females over five consecutive days). The experiments started in the dark phase (under red light conditions) and were conducted between 18:00 and 22:00. All trials were recorded with a D-link camera (DCS-3710 Day & Night WDR network camera) sensitive to red light. To avoid any effects from the presence of an observer on mouse behavior all experiments were simultaneously observed outside the experimental room on a monitor connected to the video camera system. Later, recorded videos were analyzed using the program Noldus Observer XD 9.0 to verify initial partner preference of females based on direct monitor observations.

**Figure 3:**
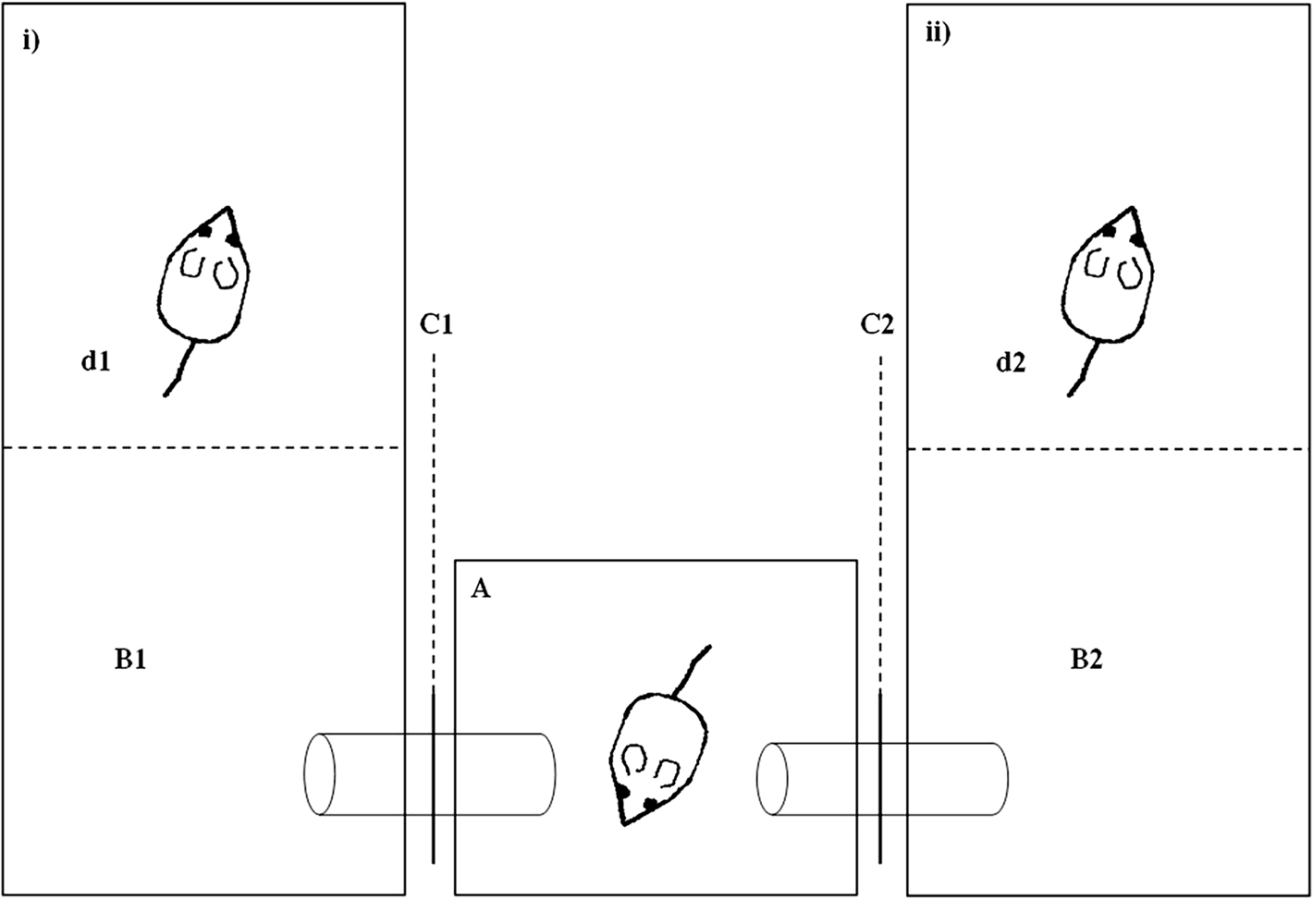
**Social preference test.** The central cage **(A)**, where the female was located, was connected with the two male cages **(B1, B2)** by plastic tubes. The tubes were closed at the beginning of the experiment, after acclimatization the barriers were removed **(C)** to enable females **(A)** to access the two males’ cages. Males cages were separated into two compartments using perforated plastic dividers **(d1, d2)**, which allowed olfactory, acoustic and visual inspection between sexes, however preventing a mating event. Each male was restrained to only one compartment of their cage **(i, ii)**.

The experimental apparatus consisted of three cages (Figure 3): one central female cage (26.5 × 20.5 × 14 cm) was connected with plastic tubes to two male cages on the left and right side, each measuring 36.5 × 20.5 × 14 cm (Figure 3). Male cages were separated into two compartments (i, ii) using an acrylic glass divider with several holes. This setup allowed exchange of acoustic, tactile and odour signals, however prevented mating. First, at the beginning of the experiment males were placed individually into their compartments (i), and bedding material from the male’s original cage was scattered in the other compartment (ii) of the male cages to provide direct odour cues for the female (Figure 3). Afterwards females were placed in the central cage. The connecting tubes were closed with regular inflated balloons to prevent the female from 1) entering male cages until the habituation period for all animals ended and 2) obtaining odour cues from male’s bedding material before the experiment started. After 10 minutes of acclimatization, video recording started and the balloons were simultaneously deflated to make the tubes (and cages) accessible for the female. The female was considered to have entered or left a cage when its nose was visible in the cage. The social preference recording began once the female had visited both male cages. This procedure ensured that the female was aware of both males (when she left the second male’s cage). Each trial lasted for 10 minutes, which has been shown to be a good predictor for partner preference [9]. Time the female had spent in each male cage was recorded by using a stopwatch. A male was classified as ‘preferred’ if the female spend more than 60% of the time in its compartment [following 9]. Subsequently, half of the females were paired with their preferred (P), the other half with their non-preferred (NP) male in a new cage and returned to their housing room. Females that were paired with P or NP males did not differ in weight (Mann-Whitney U test: U = 43, P = 0.631, r = 0.11). All pairs were separated after five days. The litter sizes, individual F2-pup weight and sex ratio were determined at weaning.

### *Salmonella* infection

To assess pathogen clearance of F2 offspring from P versus NP sires, a total of 72 offspring resulting from 23 families were experimentally infected on two consecutive days. The number of individuals was balanced for partner preference (P/NP) and sex. All mice were adults (15-21 weeks old) and received an intraperitoneal (IP) infection of 200 μl *Salmonella enterica* serovar Typhimurium (10^3^ cfu mL^-1^, strain LT2). The bacteria (stored as slants at 4°C and originated from frozen stocks at -80°C) were cultured in 7.5 ml of heart-brain infusion at 37°C for 13 h (overnight) while shaking at 170 rpm. The overnight solution was diluted with sterile phosphate buffered saline (PBS) until the desired dilution of 10^3^. To verify the concentration, serial plating of the dilution (50 μl per plate and three plates per solution) was performed.

All animals (N = 72) were divided into three groups to obtain a sample size of 24 individuals per week during the experimental period of three weeks. Groups were balanced for partner preference and sex. Individuals were euthanized and dissected over three weeks: group 1 after seven days (N = 24), group 2 after 14 days (N = 24), and group 3 21 days post infection (N = 24). The health condition of mice was examined daily until the end of the experiment. All animals were weighed weekly and euthanized humanely using an overdose of CO_2_. Additionally, we also monitored the survival of additional P (N = 64) and NP (N = 44) offspring in the colony (same age class), which were not experimentally infected. To determine pathogen loads of experimentally infected animals, spleens of euthanized animals were immediately removed and homogenized (Dispergierstation, T 8.10, IKA®-Werke) in 1 ml PBS under sterile conditions. Afterwards, 50 μl of each homogenate was plated on selective agar plates (Önöz Salmonella agar Merck, Darmstadt, Germany), and incubated overnight for 18 hours at 36°C. Pathogen loads per spleen were determined by calculating bacterial concentration (cfu/spleen) of spleen homogenates [25,37] using the mean of three replicate plates. The experimental procedure was in accordance with ethical standards and guidelines in the care and use of experimental animals of the Ethical and Animal Welfare Commission of the University of Veterinary Medicine Vienna (Permit No. BMWF-68.205/0261-II/3b/2011).

### Statistical analyses

To assess differences in female reproductive success when paired with P versus NP we compared the number of litters produced in the different treatments using a χ2 test. Differences in litter sizes were analysed using a general linear model (GLM) with pairing (P or NP male) as a fixed factor and female body mass as a covariate. Mean pup weight (offspring quality) was calculated as the total weight of litters divides by litter size at weaning. We compared mean pup weight of litters sired from P versus NP males in using a GLM with pairing (P or NP male) as a fixed factor and female body mass as a covariate. Sex ratio was analysed using a generalized linear model (GZLM) with a binomial distribution and a logit link function. The number of males within a litter was included as the dependant variable and litter size as binomial denominator. Pairing (P or NP male) was integrated as a fixed factor and female body mass as a covariate. These statistical analyses were performed using IBM SPSS® version 19 (SPSS Inc., Chicago, Illinois) software.

For subsequent pathogen and survival models we tested whether initial body mass was randomly distributed across experimental groups by using a linear mixed-effects model (LMM) with offspring sex and partner preference (P, NP) as fixed effects and week (time to euthanasia, see above) as a covariate. We also entered the day of experimental infection as a random effect to control for statistical non-independence of trials started on one or the other date. Additionally, to control for sex difference in body mass, we calculated residuals of body mass on sex and used these residuals as sex-independent measure of body mass. We transformed individual body mass and count data (pathogen load) using log-transformation to enhance normality and homogeneity of variances.

To assess individual pathogen load, we applied a LMM, entering partner preference (P/NP), and sex as fixed effects, week as a covariate and starting date of the experiments and family (N = 23) as random effects. We analysed survival using a generalized linear mixed-effects model (GZLMM) with binomial error distribution, entering partner preference treatment, and sex as fixed effects, week and body mass as covariates and starting date of the experiments and family ID as random effects. Statistical analyses were performed using ‘R’ (version 2.14.1). We implemented LMMs using the ‘lme’ function of the ‘nlme’ package, and GZLMM using the ‘glmmPQL’ function of the ‘MASS’ package.

We included all two-way interactions into initial models and applied a backward stepwise removal procedure [63] to avoid problems because of inclusion of non-significant terms (P < 0.05) [64]. Removed variables were re-entered one by one to the final model to obtain relevant statistics.

## Competing interests

The authors declare that they have no competing interests.

## Authors’ contributions

SR and DJP designed the study, SR, SS, KET, MT and FW collected the data, SR and AH performed the analysis. All authors contributed towards writing the manuscript and approved its final version.
